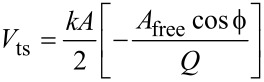# Probing viscoelastic surfaces with bimodal tapping-mode atomic force microscopy: Underlying physics and observables for a standard linear solid model

**DOI:** 10.3762/bjnano.5.176

**Published:** 2014-09-26

**Authors:** Santiago D Solares

**Affiliations:** 1 Department of Mechanical Engineering, University of Maryland, College Park, MD 20742, USA; 2 Department of Mechanical and Aerospace Engineering, George Washington University, Washington, DC 20052, USA

**Keywords:** amplitude-modulation, bimodal, dissipation, frequency modulation, multi-frequency atomic force microscopy, viscoelasticity, standard linear solid

## Abstract

This paper presents computational simulations of single-mode and bimodal atomic force microscopy (AFM) with particular focus on the viscoelastic interactions occurring during tip–sample impact. The surface is modeled by using a standard linear solid model, which is the simplest system that can reproduce creep compliance and stress relaxation, which are fundamental behaviors exhibited by viscoelastic surfaces. The relaxation of the surface in combination with the complexities of bimodal tip–sample impacts gives rise to unique dynamic behaviors that have important consequences with regards to the acquisition of quantitative relationships between the sample properties and the AFM observables. The physics of the tip–sample interactions and its effect on the observables are illustrated and discussed, and a brief research outlook on viscoelasticity measurement with intermittent-contact AFM is provided.

## Introduction

Atomic force microscopy (AFM) has developed considerably since its introduction in the mid-1980s, and today constitutes one of the most powerful and versatile tools in nanotechnology [1–3]. Besides topographical imaging, it is also commonly used to map conservative and dissipative interactions across nanoscale surfaces, from which compositional contrast can be inferred. For soft samples the contrast is often associated with viscoelasticity for which measurements are most commonly carried out by using contact resonance techniques [4–8], whereby classical properties are approximated by using contact models under small-amplitude oscillatory deformations. Such characterization is much more challenging to carry out by using intermittent-contact techniques due to the non-linear behavior of the probe–sample forces, although significant progress has already been achieved by using multi-frequency methods [9]. The contact models used so far are not true viscoelastic models, since they do not exhibit time-dependent stress and strain relaxation, but they have been shown to be applicable and useful for certain types of samples [9]. The purpose of this paper is to explore computationally the expected physics and the response of the observables for a viscoelastic contact model that exhibits both creep compliance and stress relaxation. Thus, the standard linear solid model (SLS [10]) is used and its complexities and non-idealities are simulated within bimodal AFM, which has become a popular multi-frequency method since its introduction ten years ago [11–12]. The SLS is a simple model that does not fully reproduce the behavior of true surfaces, but since it exhibits the correct qualitative behavior for creep compliance and stress relaxation, its study can highlight the range of open issues that remain in the development of surface viscoelasticity measurement methods based on intermittent-contact AFM. This paper begins with a background section providing a very brief description of multi-frequency AFM, a summary of previous viscoelastic characterization works, a brief introduction to a few viscoelasticity models previously used in AFM and a brief discussion on non-viscoelastic dissipative interactions. The background section is followed by the most relevant results for single-mode and bimodal tip–sample interactions, including force trajectories and discussions on the key observables, after which a brief discussion and a conclusions section are offered. Finally, a short section describing the simulation methods is provided.

## Background

### Multi-frequency atomic force microscopy – multimodal operation

Multi-frequency AFM [13] is a family of techniques in which the cantilever probe is driven simultaneously at more than one frequency with the purpose of expanding the amount and type of information that can be acquired during each scan. Most commonly this is accomplished by driving simultaneously more than one cantilever eigenmode (multimodal characterization), such that the contrast signals from each eigenmode serve different purposes. For example, within the first multi-frequency technique, proposed by Rodriguez and Garcia in 2004 [11–12], the fundamental cantilever eigenmode is driven by using the conventional amplitude-modulation scheme (AM-AFM, tapping-mode [2]) to obtain the topography, while the second eigenmode is excited with constant drive amplitude and frequency. Compositional contrast is extracted from the response amplitude and phase of the second eigenmode (in this paper this mode of operation is referred to as AM-OL since the first mode is driven by using amplitude-modulation and the second mode is driven in ‘open loop’). Since the settings of the higher eigenmode are not controlled by the AM-AFM loop, its excitation amplitude (and in principle also the drive frequency) can be adjusted almost at will to explore a wider range of interactions with high sensitivity. There currently exists a variety of other multi-frequency techniques that use multi-eigenmode excitation with two or three drive signals [9,11–12,14–20], discrete multi-frequency excitation for a single eigenmode [21–22], band excitation single- or dual-mode characterization [23–24] and techniques based on the observation of higher harmonics and their inversion to obtain force distance curves [25–26]. Most of the discussion in this paper is based on the AM-OL method of Rodriguez and Garcia [11–12], which is the most common, but some of the discussion is also applicable to bimodal methods involving frequency-modulation (FM-AFM [3,9,18,27].

### Characterization of viscoelastic surfaces with AFM

Viscoelastic characterization is generally performed with contact-mode-based methods involving a sinusoidal displacement of the sample or the cantilever. One of the oldest reports is the force modulation method [28], whereby the sample is driven sinusoidally, causing an analogous response in the cantilever, such that its oscillation amplitude and phase can be used to approximately calculate the sample storage and loss moduli. Similarly, in contact resonance methods [4–8] the user generally measures the cantilever frequency response to small amplitude excitations, from which an effective resonance frequency and quality factor can be computed and post-processed to also give the storage and loss moduli. These moduli are classical bulk quantities, but AFM measurements can exhibit relatively good correlation with results obtained from bulk measurements [6–7]. Analogous measurements can be performed by using the band excitation method [23,29], within which the cantilever is driven in contact mode by using a band of frequencies such that the Fourier transform of the tip response can be fit to a Lorentzian curve that readily yields the effective resonance frequency and quality factor, which in turn yield the desired moduli. A third method that provides similar observables is the dual-amplitude resonance tracking technique [6–7,21], in which the cantilever is also driven in contact mode, but by using only two sinusoidal excitations around the effective resonance frequency.

In recent years, intermittent-contact methods have also been used to gain understanding of the conservative and dissipative tip–sample interactions, simultaneously while topographical imaging is being carried out, although direct mathematical relationships between the observables and actual viscoelastic properties have, in general, not yet been developed. The main obstacles have generally been the non-linear behavior of the tip–sample forces and the non-ideal shape of the tip trajectory during impact, both of which make mathematical analyses extremely difficult (these complexities are further discussed in the Results section). In amplitude modulation AFM (tapping-mode, AM-AFM), Cleveland et al. [30] and Garcia et al. [31] proved mathematically that when tip–sample energy dissipation is absent, the phase shift remains unchanged even if the elastic properties of the sample are non-uniform across the surface. This enables the user to directly map variations in energy dissipation based on the phase contrast. In addition, one can more rigorously describe the conservative and dissipative interactions by quantifying them in terms of the virial (*V*_ts_) and average dissipated power (*P*_ts_), for which equations have been previously published for use within amplitude- [30,32–33] and frequency-modulation [34–36] methods. Within multi-frequency AFM, Lozano et al. analyzed the behavior of *V*_ts_ and *P*_ts_ for the original bimodal AFM method, which uses an open loop drive to excite the higher eigenmode [32,37]. Naitoh and coworkers reported bimodal experiments by using FM-AFM to drive both eigenmodes, in order to simultaneously acquire the topography and quantify the elasticity of a Ge(001) surface with high resolution [17]. Li and coworkers used a bimodal method in which the first eigenmode was driven by using the phase modulation scheme and the higher mode was driven in open loop, which allowed them to obtain images of the sample topography, energy dissipation and elasticity of polymer surfaces immersed in a liquid environment [16]. We have also reported experiments in which images of *V*_ts_ and *P*_ts_ were compared for different control schemes applied to the higher mode, including open loop, constant-excitation FM-AFM and constant-amplitude FM-AFM [27]. Even more recently Herruzo et al. [9] succeeded for the first time in inverting the conservative tip–sample interaction force curve along with a depth-dependent, direction-independent tip–sample dissipation coefficient by using bimodal FM-AFM with constant amplitudes for both eigenmodes.

There also exist methods for the real-time acquisition of force curves, from which conservative and dissipative interactions can be studied. Specifically, the spectral inversion method, originally introduced by Stark et al. [25] and later improved by Sahin et al. by using T-shaped cantilevers [26] uses the spectral response of one of the cantilever eigenmodes (the first torsional mode in the method of Sahin et al.) to invert the force curve without making any assumptions about the tip–sample contact model. The method has been demonstrated extensively on soft samples, but becomes subject to low-signal-to-noise ratio limitations as the sample becomes stiffer [38]. Finally, the peak-force AFM method [39], a hybrid between contact- and intermittent-contact AFM, also measures the tip–sample force in real time during approach and retract of the tip by modulating the cantilever base position above the sample with a large amplitude and with a frequency that is much lower than the fundamental frequency. This method also has the advantage that no assumptions need to be made about the tip–sample force model, although it is limited in the range of tip-velocities that can studied and, since it is a deflection-based measurement, it may be limited by low signal-to-noise ratio when small displacements or subtle features in the force curve are being studied.

### Viscoelasticity models and the standard linear solid

**Figure 1:**
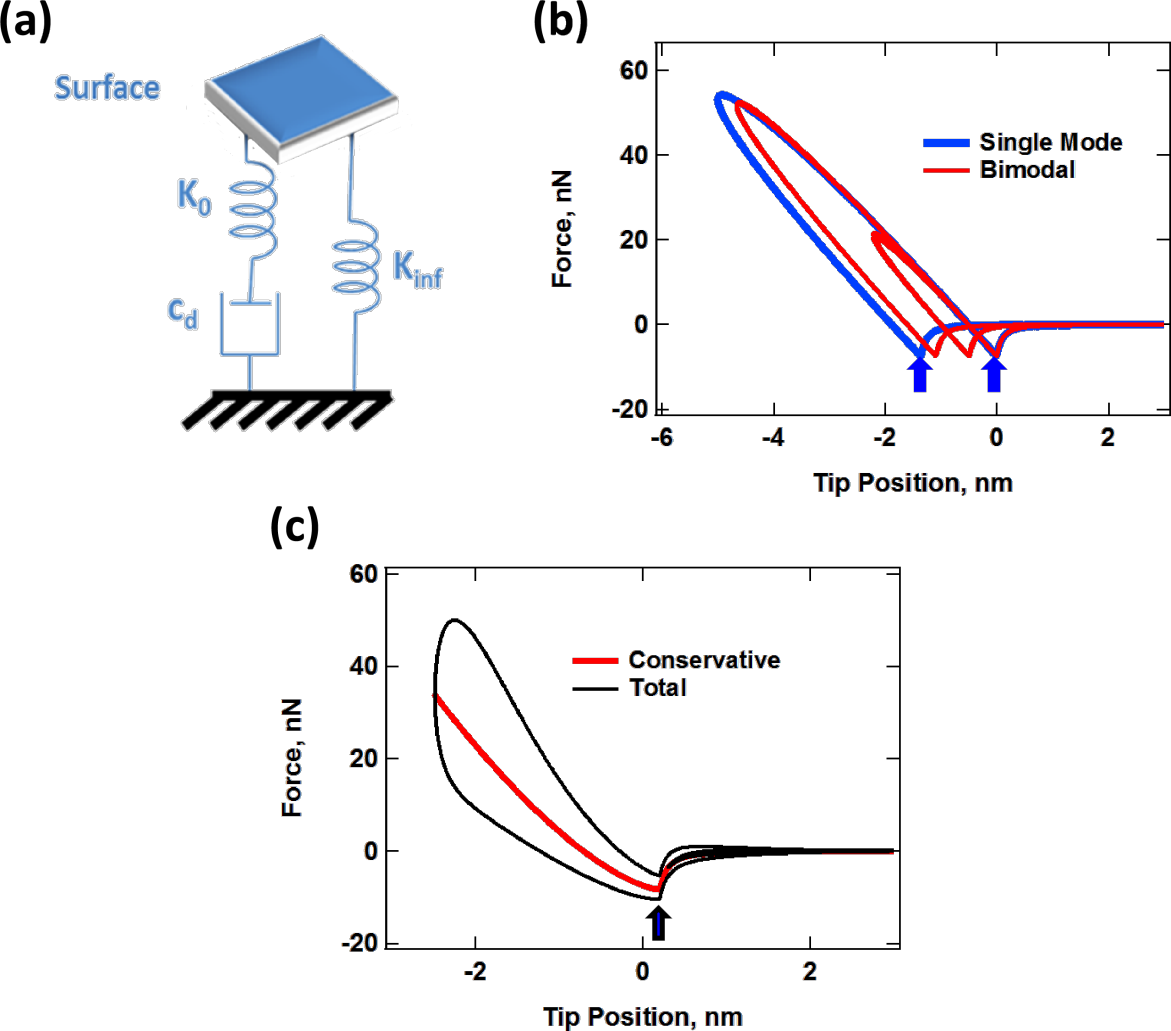
(a) Standard linear solid (SLS) model [10]; (b) simulated tip–sample force trajectories for single- and dual-mode (bimodal) AFM impacts by using the SLS. The key difference between the single- and dual-mode interactions is the possibility of multiple impacts as well as the variation in the shape of successive impacts in the latter [41–42]. The horizontal distance between the two blue arrows indicates the distance that the surface relaxed during the tip–sample contact time for the single-mode case. (c) Simulated tip–sample force trajectory by using a Hertzian contact model with a depth-dependent dissipation coefficient [34,42]. For all force curves shown, the trajectory around the dissipation loops proceeds in the counterclockwise direction.

Viscoelasticity models are used to relate the observables and calculated quantities from the AFM measurement (frequency, phase, amplitude, quality factor, etc.) to the surface properties. In contact resonance typically the Kelvin–Voigt model [40] is used, which consists of a linear spring in parallel with a damper (dashpot). It is incorporated into the solution of the cantilever equations of motion in the form of boundary conditions at the tip [4–5]. This model can reproduce time-dependent creep compliance (time-dependent strain relaxation under a constant stress) with high accuracy, but not stress relaxation (time dependent drop in stress under a constant strain). Another model often used in the study of viscoelasticity, although not commonly used in AFM, is the Maxwell model [40] which consists of the same two elements, a linear spring and a dashpot, but arranged in series. This model reproduces well stress relaxation under constant strain but not creep compliance. Within AFM it has also been common to combine a Hertzian conservative tip–sample model with a position-dependent dissipation coefficient [9,18,34]. This is the approach recently followed by Herruzo et al. [9] to obtain analytical expressions of conservative and dissipative tip–sample interaction forces by using bimodal frequency-modulation AFM. Although the authors show that their approach can be robust for the types of surfaces studied in their work, the characterization of truly viscoelastic surfaces requires being able to capture fundamental viscoelastic behaviors, in particular stress relaxation and creep compliance. The simplest model that meets these conditions is the standard linear solid (SLS), which combines the Kelvin–Voigt and Maxwell models as illustrated in Figure 1a. Figure 1b illustrates typical tip–sample force trajectories during intermittent-contact AFM single- and dual-mode simulations, whereby hysteresis occurs due to relaxation of the surface during the time that the tip and sample are in contact. In fact, for this type of model, the surface can remain temporarily depressed as the tip retracts. This can be inferred from Figure 1b by noting that the position of the maximum attractive (van der Waals) force differs during approach and retract (see blue arrows). In contrast, Figure 1c shows that when a conservative model (e.g., Hertzian) is combined with a dissipation coefficient, there is hysteresis but the location of the force minimum does not differ for the approach and retract. As a result, such models do not fully capture viscoelasticity since the surface does not actually relax, and this has important consequences with regards to the interpretation of the dissipation mechanism. Specifically, the latter models offer a dissipative mechanism in which the tip experiences a ‘friction’ force opposite to its motion, regardless of the direction in which it travels (upward or downward). In contrast, in the SLS model, the dissipation is a consequence of the simple fact that the work done by the cantilever against the surface during the approach is greater than the work done by the surface on the cantilever during the retract (since the surface relaxes during contact, the amount of work it restores to the cantilever is less than the work received from it). Despite the attractive features of the SLS, however, it is important to note that it is too simple of an approximation to describe the behavior of a real sample under intermittent-contact AFM. For example, since it uses linear springs, it is not able to capture the curvature of the repulsive part of an elastic interaction, which is well reproduced by Hertzian models [2].

**Figure 2:**
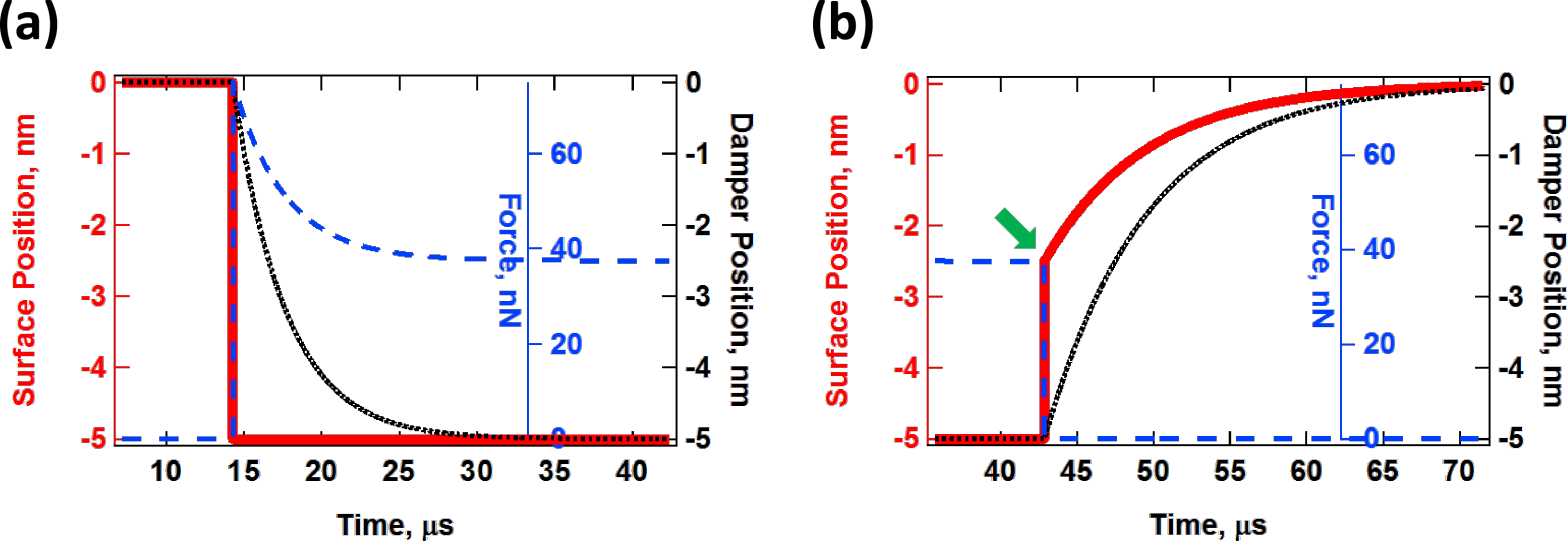
(a) Illustration of stress relaxation and (b) creep (the various traces are color coded with their respective axes). In (a) the surface is depressed and held fixed at a position of 5 nm below its relaxed state, starting at *t* = 15 μs. Following the simulation described in (a) the surface is released at *t* = 43 μs and its response is plotted in (b). The magnitude of the immediate elastic relaxation of the surface upon release, indicated by the green arrow in (b), depends on the ratio and magnitudes of the two spring constants, *K*_inf_ and *K*_0_ (see Figure 1). The simulation parameters were *K*_inf_ = *K*_0_ = 7.5 N/m and *C*_diss_ = 2.5 × 10^−5^ N·s/m.

The behavior of the SLS under the application of a constant strain is illustrated in Figure 2a. The thick red trace indicates the prescribed position of the surface, which starting at *t* = 15 μs is depressed and held at a position of −5 nm for the example shown. Immediately the damper begins to relax as indicated by the black dotted line, which results in a relaxation of the force exerted by the surface (blue dashed line). Figure 2b illustrates the behavior of the model after the surface is released, after reaching the final state in Figure 2a. Here the surface immediately relaxes elastically to a position of −2.5 nm (indicated by the position of the green arrow), and then both the position of the surface and the position of the damper gradually relax to the equilibrium state while the force acting on the surface remains at zero. These behaviors are not necessarily critical to reproduce in contact-mode, low-amplitude AFM measurements (e.g., contact-resonance characterization), but are very important in intermittent-contact AFM during which the surface undergoes rapid distortion and relaxation between and during successive interactions with the tip, and where the accurate measurement of viscoelastic properties is much more challenging to carry out [43].

### Non-viscoelatic dissipative interactions

**Figure 3:**
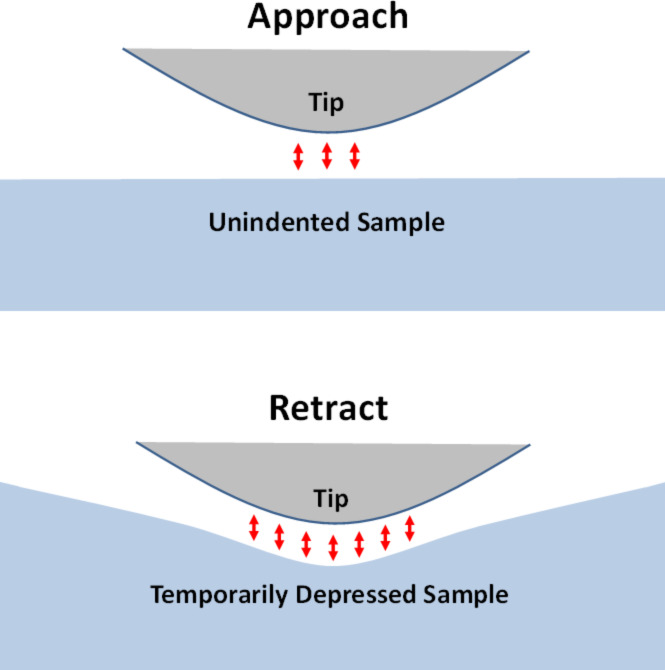
Illustration of tip–sample physical adhesion. In this case the interaction area between tip and sample is greater during the retract than during the approach, due to a change in the non-bonded attractive forces, leading to additional dissipation as the cantilever breaks free from the surface.

The present work studies an AFM tip that is interacting with a clean SLS surface, so no further interactions are included other than attractive van der Waals forces. However, in practice there can be a number of other interactions that can obscure or hinder the measurement of viscoelasticity by using intermittent-contact methods. Well-known interactions of this type include capillary forces [44], plastic behaviors [45], chemical adhesion and topographical artifacts [46] and even geometry-driven physical adhesion artifacts. As illustrated in Figure 3, if the tip indents a cavity into the surface and the surface remains temporarily depressed, the non-bonded interactions during the retract may be greater than during the approach due to the greater sample surface area that is near the tip. This, in turn, would lead to a hysteresis loop in the tip–sample force trajectory, whereby the cantilever would be required to perform additional work in order to break free from the surface. All of the above non-conservative effects influence the observables during measurements of conservative and dissipative interactions with AFM, and it is generally not possible to attribute with certainty the changes in the observables to the variation in surface material properties.

## Results

This section comprises two main sub-sections. The first sub-section provides an analysis of the tip–sample interaction physics for ideal (prescribed) and numerically computed trajectories of the tip, both for single-mode and bimodal AFM. The second sub-section explores the effect of the SLS model parameters on the observables as well as the prospect for carrying out compositional mapping by using average quantities, such as the virial and dissipated power.

### Physics of the tip–sample interaction for the standard linear solid model

#### Sample response to prescribed sinusoidal trajectories

**Figure 4:**
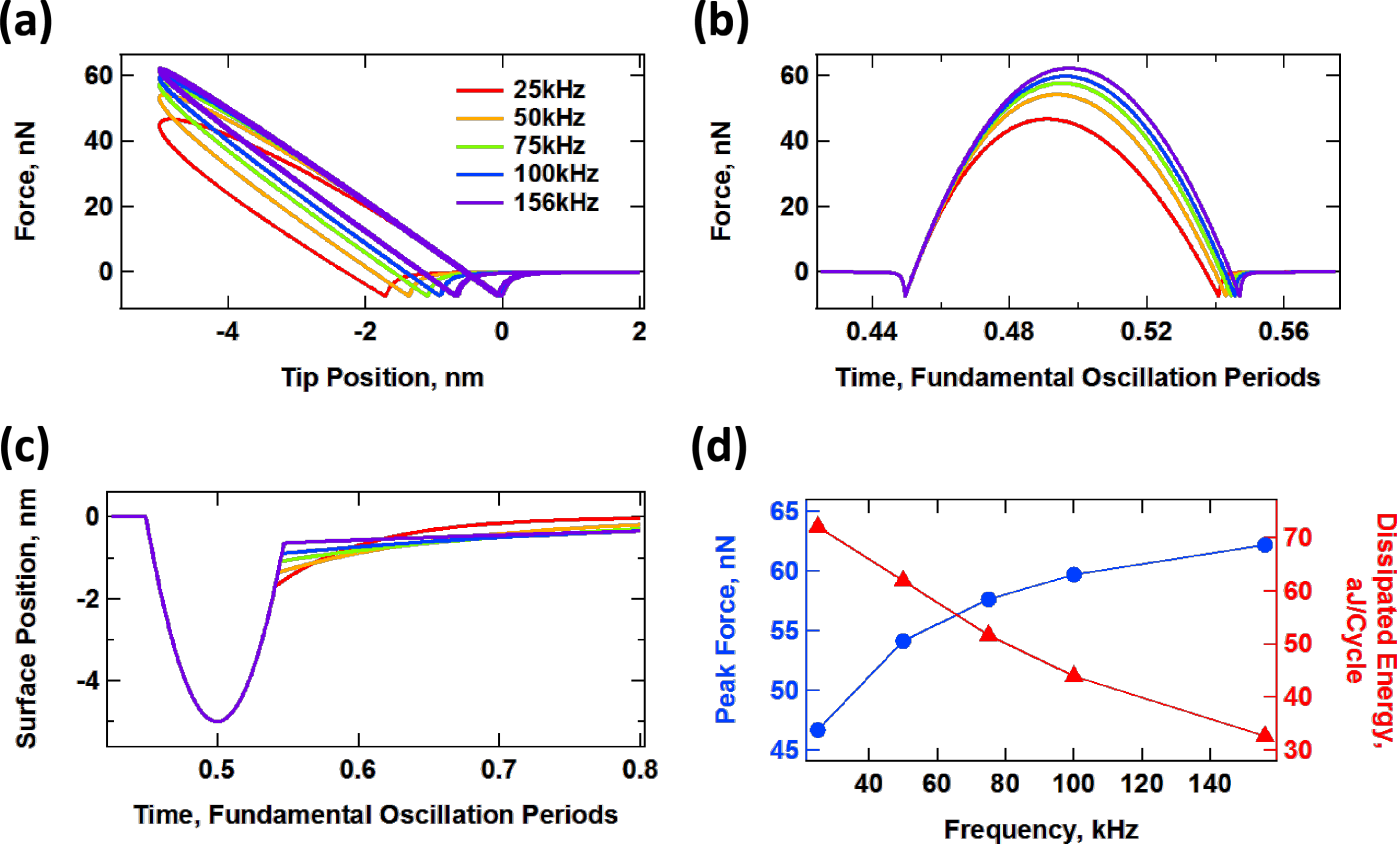
Interaction of an SLS surface with a probe oscillating along a perfectly sinusoidal trajectory given by *z*_tip_(*t*) = 95 nm + (100 nm)·cos(ω*t*), where ω is equal to 2π times the frequency indicated in the graphs (the relaxed surface is initially located at a height of zero). (a) Force–distance tip trajectory (the trajectory proceeds in the counterclockwise direction); (b) tip–sample force vs normalized time; (c) surface recovery vs normalized time; (d) peak force and dissipated energy per cycle vs cantilever frequency (the two traces are color coded with their respective axes). The SLS parameters were *K*_inf_ = *K*_o_ = 7.5 N/m and *C*_diss_ = 1 × 10^−5^ N·s/m. The time axes in (b) and (c) have been normalized by the respective fundamental period for each trace.

As starting point, consider the interaction of an SLS surface with a cantilever tip that oscillates along a perfect sinusoidal trajectory. To simulate this, we prescribe that the tip moves along a path defined by *z*_tip_(*t*) = *z*_c_ + *A*·cos(ω*t*), where *z*_tip_, *z*_c_, *A* and ω are, respectively, the tip position as a function of time, the cantilever base position, the oscillation amplitude and the fundamental angular frequency of the probe. If the SLS is relaxed in real time as the tip oscillates, one obtains results similar to those shown in Figure 4. In the first panel (Figure 1a) are depicted tip–sample force trajectories for different cantilever frequencies, ranging from 25 kHz to 156 kHz, which covers a ratio of frequencies of about 6.25, similar to the ratio of the first two eigenfrequencies of a rectangular cantilever. Figure 4b shows similar information, but plotting the force as a function of the time (notice how the contact time changes due to relaxation of the surface). Figure 4c shows the surface trajectory, including its recovery upon tip retract, and Figure 4d plots the peak repulsive force and the average energy dissipated through the hysteresis loop in the force curve over several cycles. In Figure 4b and Figure 4c the time axis has been normalized by the respective cantilever period. As expected from a viscoelastic system, the amount of surface relaxation (distance between the force minima for a given frequency in Figure 4a) decreases with increasing frequency as the hysteresis loop area also decreases (see Figure 4d). Additionally, the peak force increases. This is because at faster time scales the system behavior becomes more elastic and less viscous. This frequency dependence is an important consideration during a real experiment in which different regions of a viscoelastic sample may have different and multiple relaxation times. For some cantilever frequencies certain regions may not exhibit a measurable viscous behavior, and for other frequencies, regions of different properties may exhibit similar dissipation behaviors. As it can also be inferred from the results presented here, it is not possible to express the tip–sample force curve analytically, since it depends on the sample deformation timescale as well as on the previous history of the surface. The frequency dependence also raises an important issue with regards to the controls schemes used in bimodal AFM. It has been previously found that in order to properly compare the compositional mapping of different regions of the sample it is necessary that the higher eigenmode amplitude remains constant, which requires the use of a constant-response-amplitude method such as constant-amplitude frequency-modulation [27]. However, if the frequency shift is significantly different for different regions, according to the results of Figure 4a, they may not be directly comparable since the interplay of conservative and dissipative interactions may be artificially changed in a different way for each region.

#### Sample response to prescribed bimodal trajectories

**Figure 5:**
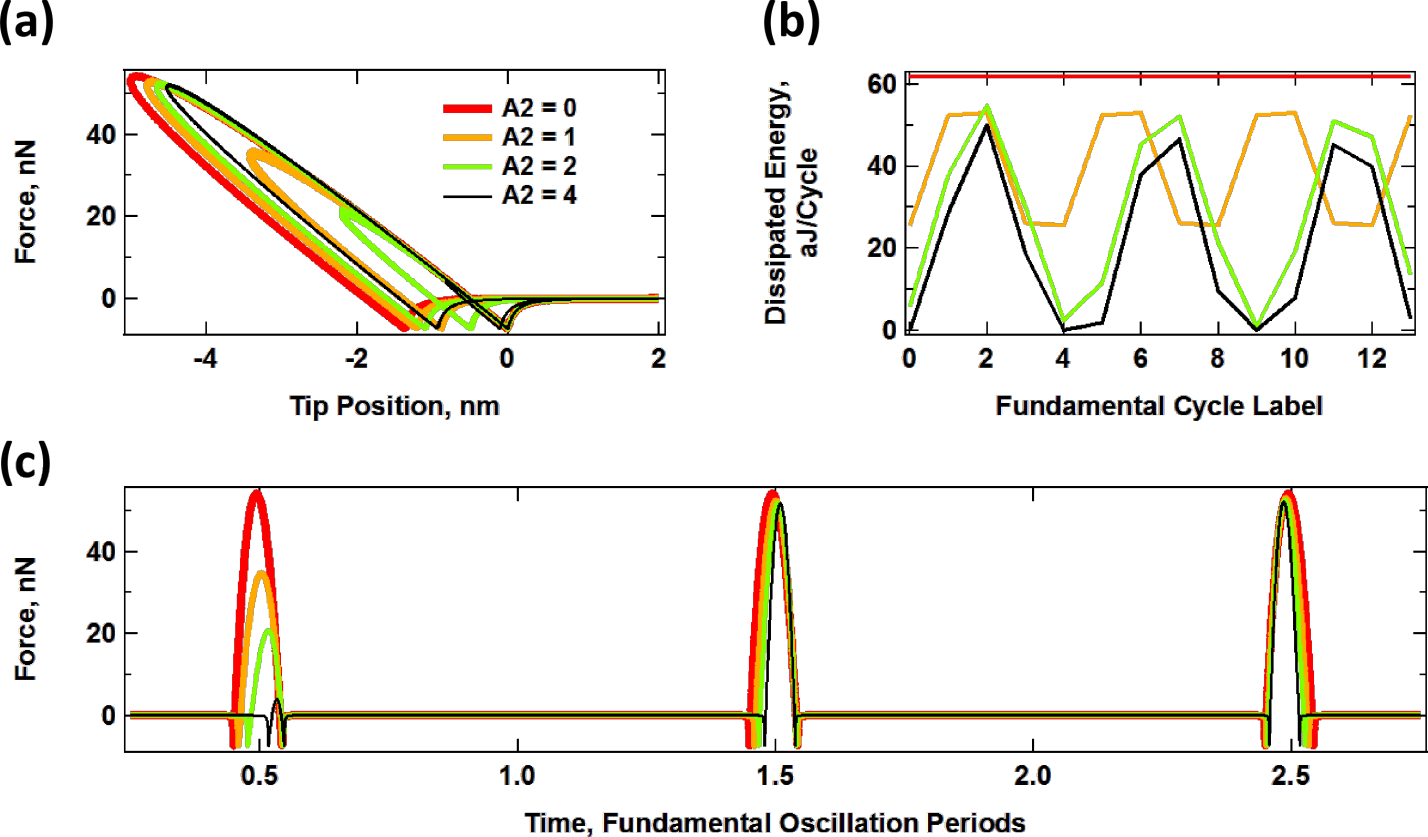
Interaction of an SLS surface with a probe oscillating along a bimodal trajectory of constant maximum penetration, given by *z*_tip_(*t*) = (95 nm + *A*_2_) + (100 nm)·cos(ω_1_*t*) + *A*_2_ cos(6.21·ω_1_*t*), with ω_1_ = 2π (50 kHz), *K*_inf_ = *K*_o_ = 7.5 N/m and *C*_diss_ = 1 × 10^−5^ N·s/m. (a) Force–distance trajectories for varying *A*_2_; (b) dissipated energy per cycle for successive fundamental cycles and varying *A*_2_; (c) example of successive tip–sample impacts for the same cases. The color code is the same for all plots and the unit of *A*_2_ is nm.

**Figure 6:**
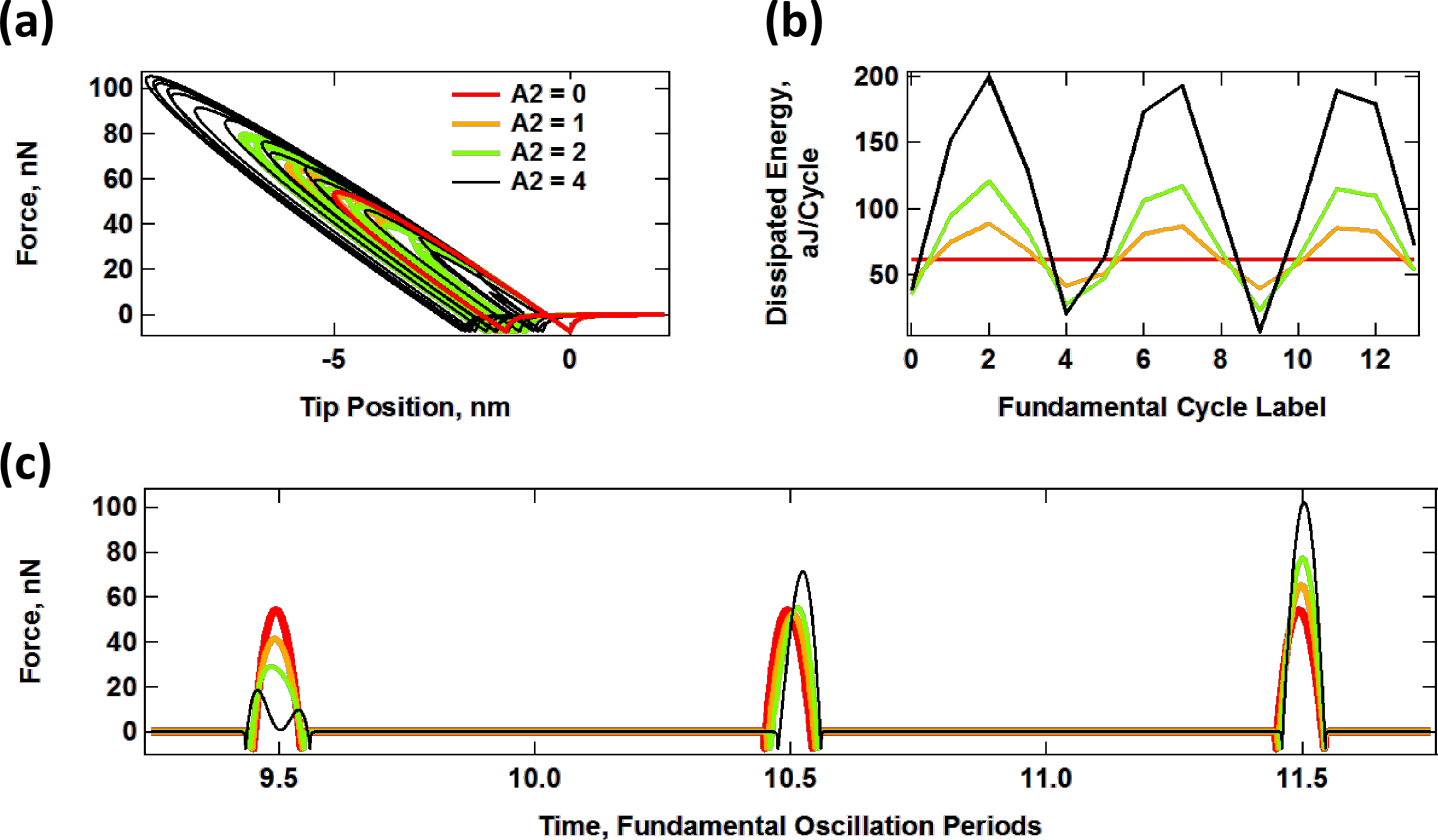
Interaction of an SLS surface with a probe oscillating along a prescribed bimodal trajectory of variable maximum penetration, given by *z*_tip_(*t*) = 95 nm + (100 nm)·cos(ω_1_*t*) + *A*_2_·cos(6.21·ω_1_*t*), with ω_1_ = 2π (50 kHz), *K*_inf_ = *K*_o_ = 7.5 N/m and *C*_diss_ = 1 × 10^−5^ N·s/m. (a) Force–distance trajectories for varying *A*_2_; (b) dissipated energy per cycle for successive fundamental cycles and varying *A*_2_; (c) example of successive tip–sample impacts for the same cases. The color code is the same for all plots and the unit of A_2_ is nm.

For the simulated interaction of an SLS surface with a tip oscillating along a prescribed bimodal trajectory of the form *z*_tip_(*t*) = *z*_c_ + *A*_1_·cos(ω_1_*t*) + *A*_2_·cos(ω_2_*t*) we are interested in studying the system response as the amplitude of the second eigenmode changes. One can envision that for small values of *A*_2_, which are smaller than the distance that the sample penetrates into the surface, the SLS will interact with a trajectory having more than one oscillation loop (since the tip will travel up and down according to the second frequency oscillation while still remaining under the sample for a contact time dictated by the first oscillation frequency). However, as *A*_2_ increases and exceeds the penetration depth, the surface will only interact with single-loop oscillations where the tip will dip into and come out of the sample at a frequency dictated by the second oscillation frequency. More than one impact will be possible for every fundamental cycle because the period of the second oscillation is smaller than that of the first oscillation (for the example shown here the ratio of eigenfrequencies was set to about 6.21 in order to mimic the first two modes of a rectangular cantilever). As a result, measurements with different levels of penetration and with different amplitude values and ratios may not be directly comparable. Furthermore, in carrying out this ideal comparison of perfectly controllable sinusoidal tip oscillations, there are two extreme behaviors that need to be considered as *A*_2_ increases. First, one could prescribe that the maximum tip–sample penetration remains the same in all cases, such that any increase in the value of *A*_2_ is compensated by an equal increase in the cantilever position, *z*_c_. Alternatively, one could prescribe that the cantilever position remains the same in all cases, such that greater a penetration will be achieved as *A*_2_ increases (as discussed below, the real situation lies in between these extremes, unless FM controls are used with which the penetration is controllable [9]). Figure 5 shows the results for the former case, and Figure 6 shows the results for the latter case. For each figure, panel (a) shows force distance trajectories for different values of *A*_2_ (including *A*_2_ = 0) and panel (b) shows the energy dissipated during successive tip–sample impacts (this dissipation was calculated by integrating numerically the area of the dissipation loops), and panel (c) shows a few examples of successive tip–sample impacts, illustrated as time-dependent forces. Interestingly, even in the case in which the peak penetration is fixed for different values of *A*_2_, the peak forces still differ considerably for successive impacts since the trajectory is bimodal. Furthermore, there are some impacts during which the interaction between the tip and the sample is almost negligible (see, for example, the first impact for *A*_2_ = 4 in Figure 5c). The dissipation loops in the force curves for the case of constant maximum depth (Figure 5a) show a gradual transition from a low frequency response to a high frequency response, qualitatively similar to what is observed for single-eigenmode oscillations (Figure 4a), but with irregular impacts. As *A*_2_ increases, the dissipation loops are dominated more and more by the frequency of the second eigenmode and the average level of dissipation drops (Figure 5b). The irregularity of the impacts is easily noticeable in the irregular oscillation of the dissipated energy for successive impacts (Figure 5b), as well as in the plot describing the force as a function of time (Figure 5c). Finally, it is not surprising that the contact time also decreases gradually as the second eigenfrequency becomes more dominant (compare Figure 5c to Figure 4b). Note: the contact time for each impact can be inferred from the graphs of the tip–sample force vs time by examining the horizontal spacing between the two force minima surrounding each peak in the plots (the first of these minima occurs when the tip approaches the sample and crosses the attractive tip–sample force region and goes into the repulsive region, and the second one occurs during the retract, when the tip leaves the repulsive region and crosses through the attractive region again).

In the case of varying penetration (constant *z*_c_), the dissipation loop areas increase as *A*_2_ increases since the oscillation of the second eigenfrequency does not replace that of the first eigenfrequency, but rather their effects are additive. The effect of the low-frequency oscillation on the surface is always present to the same degree. Therefore, the average surface relaxation distance (distance between the approach and retract tip–sample force minima in Figure 6a) increases with *A*_2_, as does the average level of dissipation (Figure 6b). Similarly, the range of contact time also increases, although as expected, there is high variability for different successive impacts. For example, the contact time increases for the first impact shown in Figure 6c, but decreases for the two subsequent impacts. This is a significant difference with respect to single-mode AM-AFM.

#### Sample response to simulated bimodal trajectories

**Figure 7:**
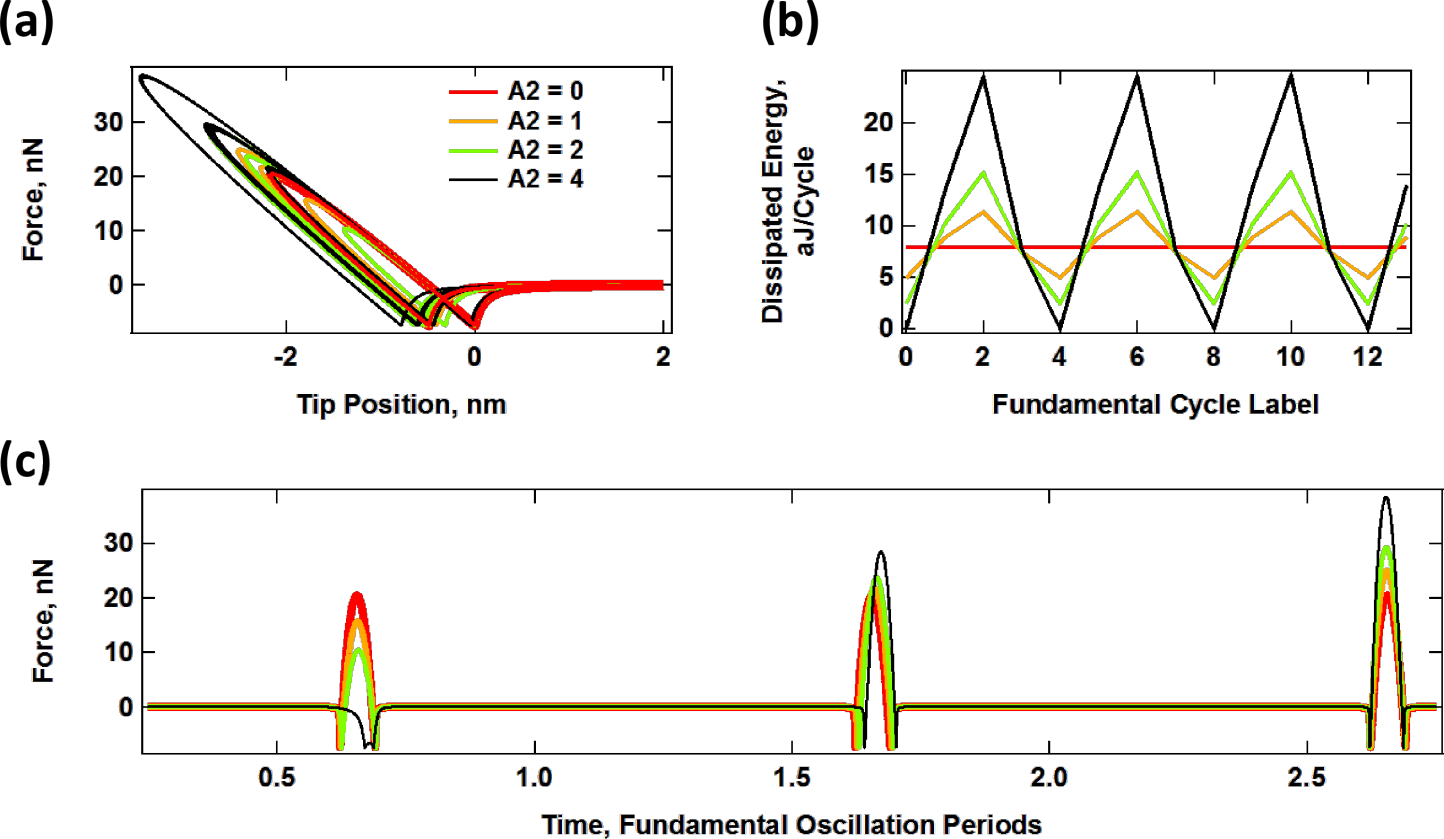
Interaction of an SLS surface with a probe oscillating along a realistic AM-OL bimodal trajectory obtained by simulating the cantilever dynamics. (a) Force–distance trajectories for varying *A*_2_; (b) dissipated energy per cycle for successive fundamental cycles and varying *A*_2_; (c) example of successive tip–sample impacts for the same cases. The first eigenmode free amplitude was 100 nm in all cases and *A*_2_ is the free oscillation amplitude of the second eigenmode (in nm). The cantilever position is *z*_c_ = 80 nm. The SLS parameters are the same as in Figure 5 and Figure 6. The eigenfrequency ratio of the first two eigenmodes is 6.25 instead of 6.21, which was used in Figure 5 and Figure 6. This different ratio was chosen to illustrate a different impact periodicity (the periodicity is 4 in this case, whereas it is 100 for Figure 5 and Figure 6). The color coding is the same in all panels.

**Figure 8:**
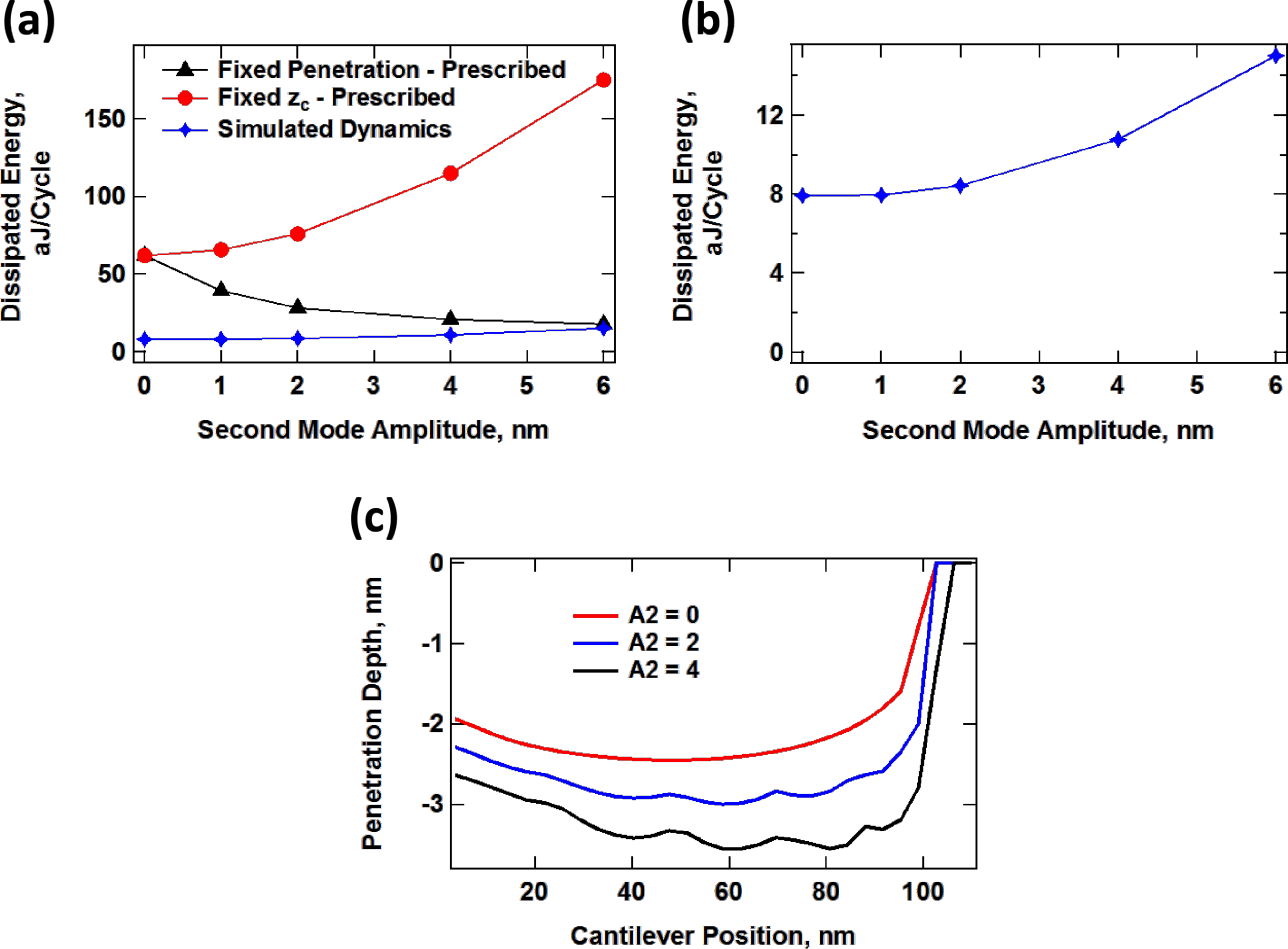
(a) Dissipated energy vs second eigenmode free amplitude for an SLS surface interacting with a tip following a prescribed bimodal oscillation with constant penetration maximum (see Figure 5), a prescribed bimodal oscillation with variable penetration maximum (see Figure 6) and a realistic dynamics trajectory solved in real time for AM-OL operation (see Figure 7); (b) expanded view of the result for the realistic dynamics trajectory (same trace as in (a) but plotted by using a different scale); (c) lowest tip position reached by the tip over 15 fundamental cycles after reaching steady state as a function of cantilever rest position during a simulated spectroscopy measurement for the realistic tip trajectory using the parameters given for Figure 7.

A direct comparison of the results for the prescribed trajectories shown in Figure 5 and Figure 6 with realistic trajectories in which the dynamics determine the tip–sample penetration is not straightforward since analytical solutions of the trajectory cannot be obtained. However, it is still possible to make some general observations. In the results of Figure 7, the dynamics of the cantilever have been solved numerically in real time, driving the first two eigenmodes at their respective natural frequencies, with *A*_1_ = 100 nm and with the values of *A*_2_ indicated on the plots. In general, the results resemble those of Figure 5 and Figure 6. There is overall lower penetration into the surface than for the prescribed trajectories despite the relatively low cantilever position of 80 nm, since the presence of the sample perturbs the cantilever oscillation [2]. Penetration increases with higher values of *A*_2_ (see Figure 7a) as previously reported [42], similar to Figure 6. The average level of dissipation for different fundamental cycles (Figure 7b) follows a more regular pattern due to the use of a slightly different ratio of eigenfrequencies for the first and second mode (see comment in the caption of Figure 7). The time-dependent force trajectories (Figure 7c) are equally rich and a wide variation in the contact time is again observed. Figure 8a compares the trends in dissipated energy as a function of *A*_2_ for the three cases analyzed in Figures 5–7. It shows that the trend for the real case lies in between the results of the prescribed trajectories with constant penetration and the prescribed trajectories of additive penetration for the two eigenmodes, while being qualitatively closer to the latter. That is, dissipation increases with *A*_2_, but in a more gradual fashion than if penetration would increase by the same amount (Figure 8b shows an expanded view of the simulated dynamics trace in Figure 8a). Finally, Figure 8c shows the peak tip penetration as a function of *A*_2_ for a simulation of a spectroscopy curve in which the cantilever is approached towards the surface. The results confirm that there is a gain in penetration as *A*_2_ increases, but the gain in penetration is smaller than the increase in *A*_2_.

**Figure 9:**
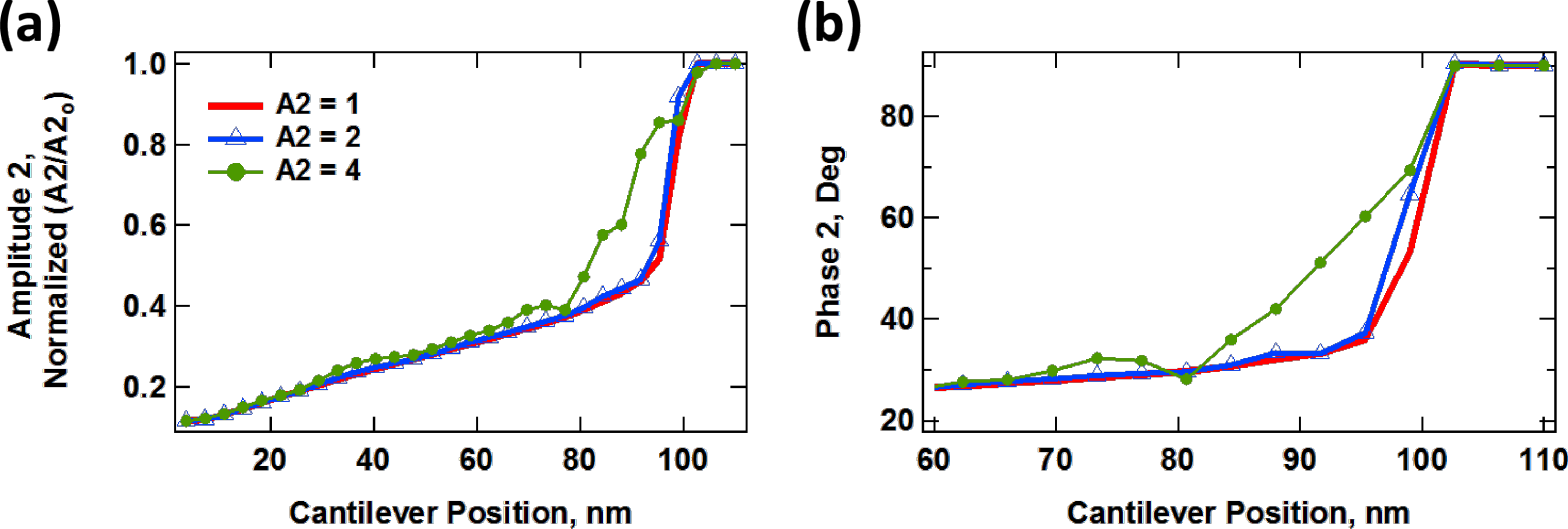
Simulated amplitude and phase spectroscopy curves for realistic cantilever trajectories calculated using parameters similar to those of Figure 7 for AM-OL operation. (a) Normalized amplitude (*A*_2_/*A*_2-free_) vs cantilever position; (b) phase vs cantilever position.

As can be inferred from the previous results, the behavior of the dissipated energy and the tip–sample forces is extremely complex and cannot be predicted a priori. In addition, the observables that are available during an AFM experiment can be quite sensitive to the imaging conditions, depending on the imaging mode used. For example, Figure 9 shows the behavior of the normalized second mode amplitude (*A*_2_/*A*_2-free_, Figure 9a) and phase (Figure 9b) for AM-OL spectroscopy curves simulated with parameters similar to those of Figure 7, for different values of *A*_2_, including the full dynamics of the first three cantilever eigenmodes. As the various traces show, the trends are not always regular nor follow a simple pattern.

### Effect of model parameters on the observables

#### Conservative and dissipative interactions for varying model parameters

**Figure 10:**
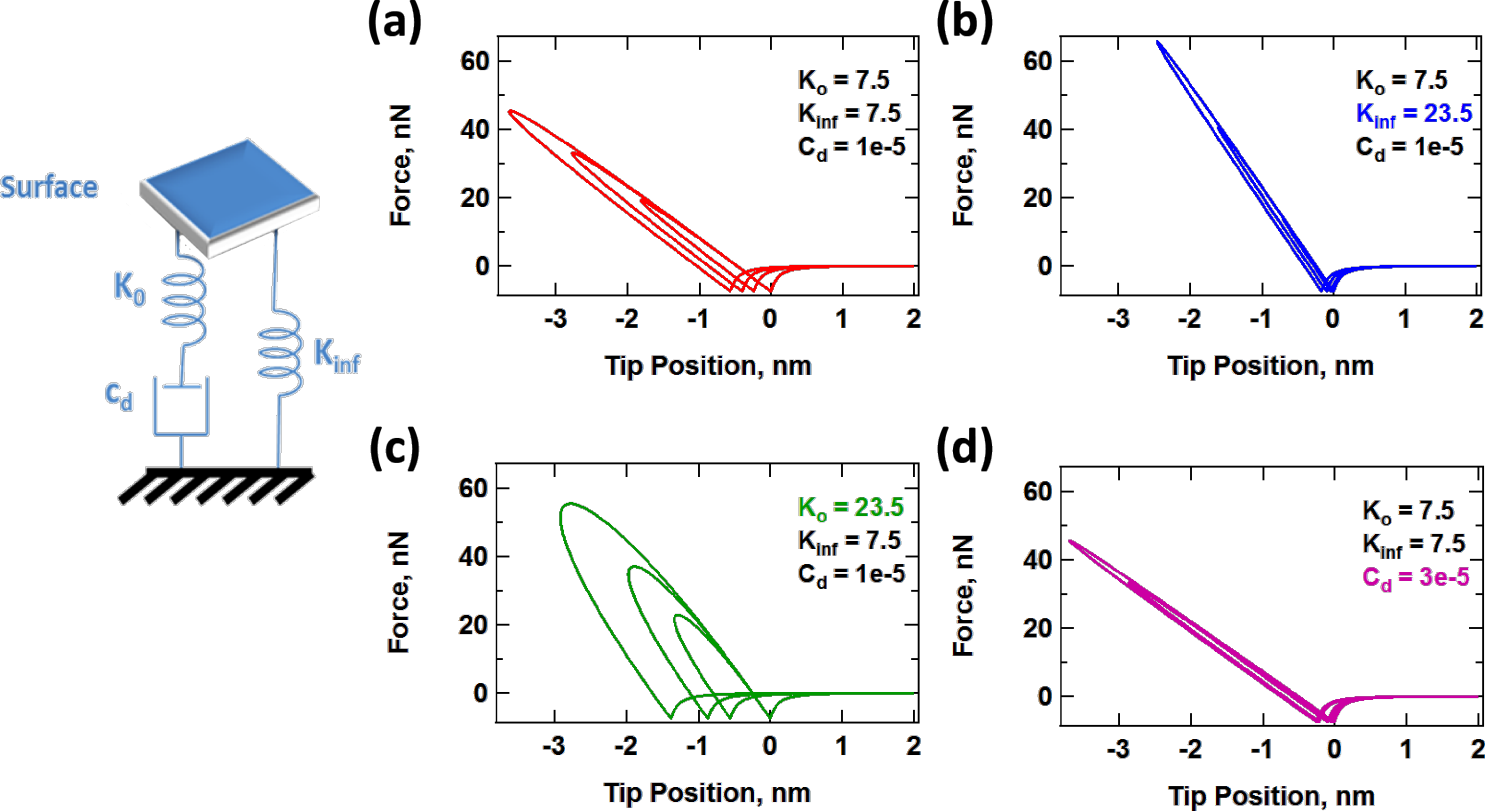
Example of the evolution of force–distance trajectories with changes in the SLS surface parameters: (a) baseline; (b) increase in *K*_inf_; (c) increase in *K*_0_; (d) increase in *C*_diss_. The parameters are similar to those given for Figure 7, except for *A*_2-free_ = 5 nm and any other parameters indicated in the figures.

In order to develop methods that recover the contact model parameters, it is necessary to understand the effect of those parameters on the probe response. To this end, Figure 10 provides an example of the changes in the force trajectory as each of the three SLS parameters is varied independently. For this example, Figure 10a serves as a baseline case and all results are plotted by using the same axis scales for easier comparison. Figure 10b shows the trajectory obtained when the stiffness of *K*_inf_ is increased to about 315% of its original value. Inspection of the SLS model (also provided in Figure 10) suggests that the spring *K*_inf_ should directly interact with the tip during tip–sample impact, and that increasing its value should result in an overall higher stiffness with smaller penetration (in general steeper force curves lead to smaller penetration for the same AFM parameters [47]). This expectation is confirmed by the force trajectory shown in Figure 10b, which also exhibits a smaller hysteresis area as a result of the shorter contact time, which is a result of shallower tip penetration. In Figure 10c the stiffness of *K*_0_ has been increased to about 315% of its original value. Inspection of the model indicates that the spring *K*_0_ interacts with the tip, but also with the damper. Increasing its stiffness results in the transmission of a larger force from the AFM tip to the damper, which should lead to a faster and greater relaxation of the latter and a correspondingly larger dissipation energy. Again, this expectation is confirmed by Figure 10c. Finally, in Figure 10d, the damper constant, *C*_diss_, has been increased to 300% of its original value, which retards its relaxation and leads to a more elastic and less viscous response with a smaller dissipation energy, as confirmed by the result.

**Figure 11:**
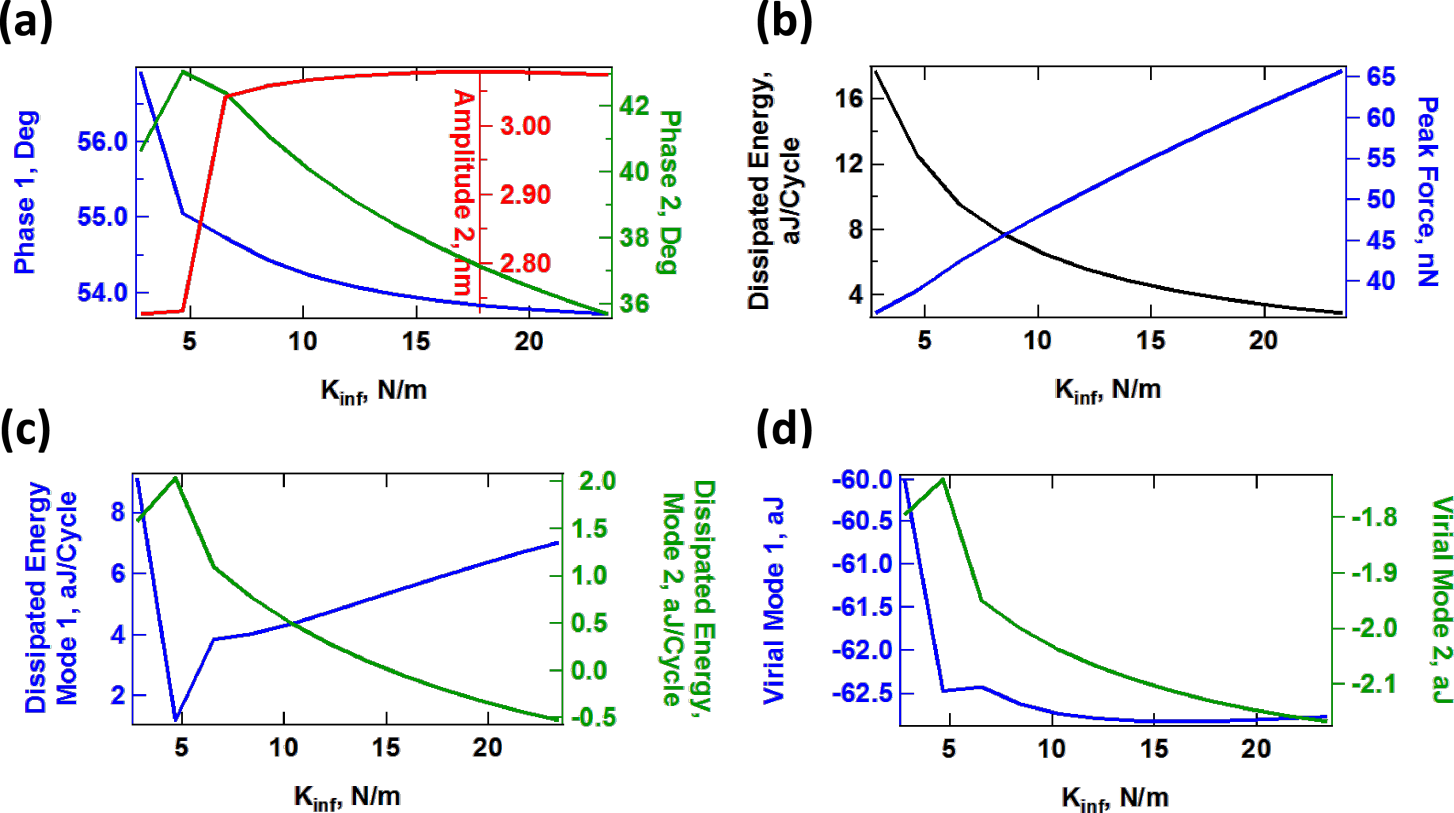
Effect of the SLS parameter *K*_inf_ on tip–sample interactions and cantilever response. (a) First and second mode phase, and second mode amplitude; (b) dissipated energy calculated from the hysteresis loop of the tip–sample force trajectory and peak impact force; (c) calculated dissipated energy from Equation 1 for the first and second eigenmodes; (d) virial for the first and second eigenmodes. Note that the peak impact force is the largest repulsive force observed over fifteen tip–sample impacts (successive impacts differ, as illustrated in Figure 7c). The parameters other than *K*_inf_ are the same used to construct Figure 10. The axes are color coded in all cases. The kinks in some of the traces correspond to transitions between dynamic regimes, in some of which the tip skips a tip–sample impact with different frequencies (for example, in one regime the tip may impact the sample every fundamental oscillation, while in another regime the tip may skip every sixth impact [42]).

**Figure 12:**
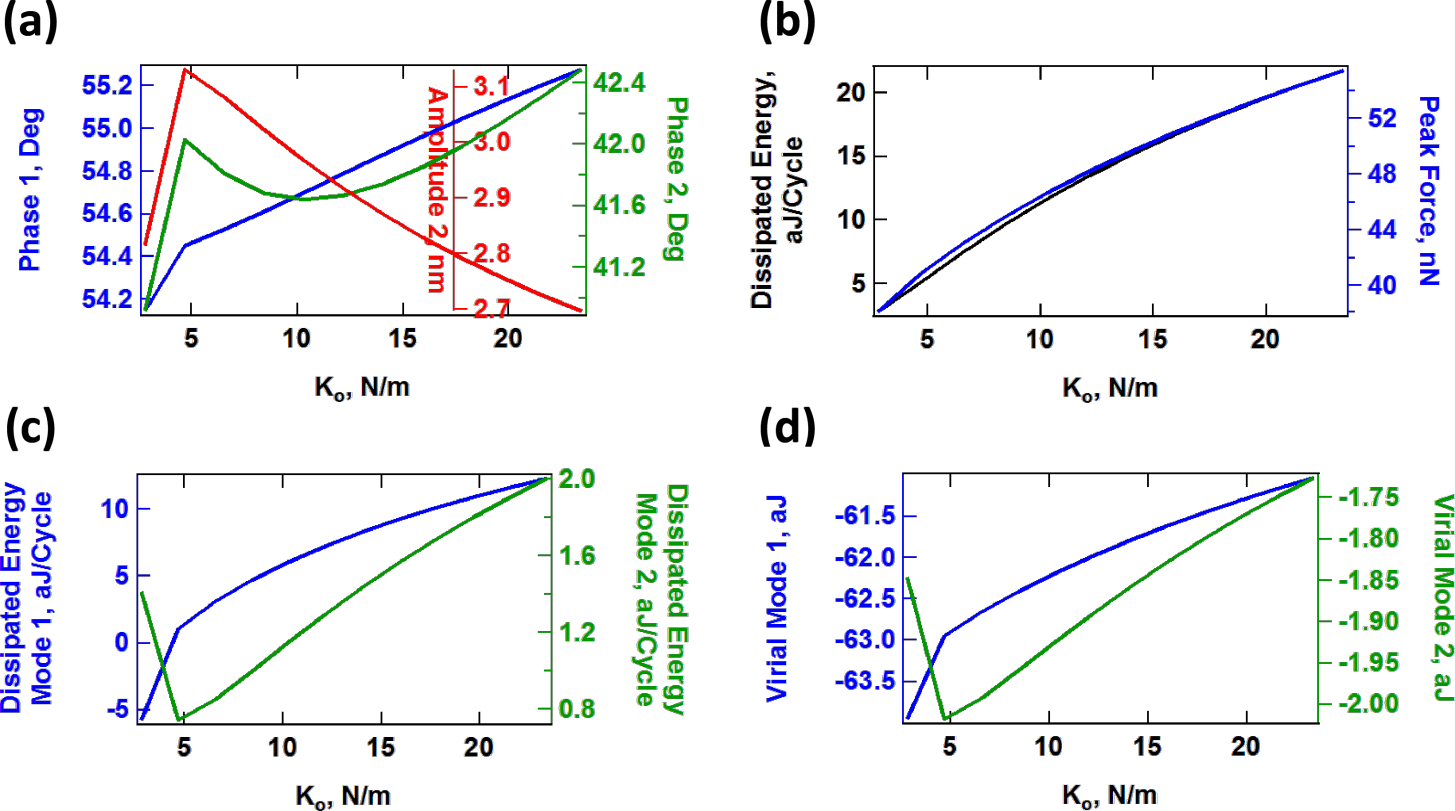
Effect of the SLS parameter *K*_0_ on tip–sample interactions and cantilever response. (a) First and second mode phase, and second mode amplitude; (b) dissipated energy calculated from the hysteresis loop of the tip–sample force trajectory and peak impact force; (c) calculated dissipated energy from Equation 1 for the first and second eigenmodes; (d) virial for the first and second eigenmodes. Note that the peak impact force is the largest repulsive force observed over fifteen tip–sample impacts (successive impacts differ, as illustrated in Figure 7c). The parameters other than *K*_0_ are the same used to construct Figure 10. The axes are color coded in all cases. The kinks in some of the traces correspond to transitions between dynamic regimes, in some of which the tip skips a tip–sample impact with different frequencies (for example, in one regime the tip may impact the sample every fundamental oscillation, while in another regime the tip may skip every sixth impact [42]).

**Figure 13:**
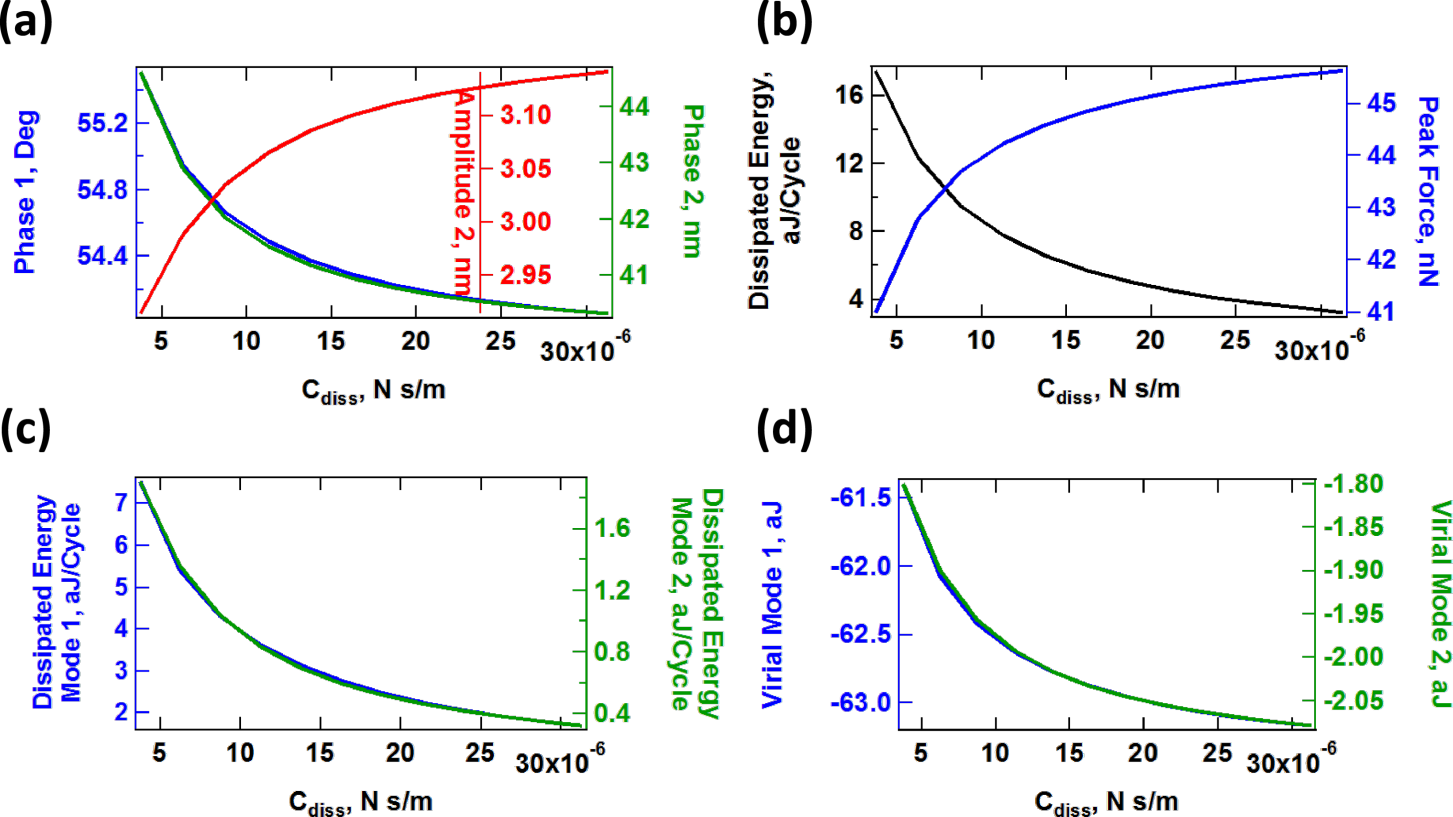
Effect of the SLS parameter *C*_diss_ on tip–sample interactions and cantilever response. (a) First and second mode phase, and second mode amplitude; (b) dissipated energy calculated from the hysteresis loop of the tip–sample force trajectory and peak impact force; (c) calculated dissipated energy from Equation 1 for the first and second eigenmodes; (d) virial for the first and second eigenmodes. Note that the peak impact force is the largest repulsive force observed over fifteen tip–sample impacts (successive impacts differ, as illustrated in Figure 7c). The parameters other than *C*_diss_ are the same used to construct Figure 10. The axes are color coded in all cases.

The above discussion suggests that one can intuitively predict the behavior of the model as its parameters change. However, the situation is more difficult in an experiment since the number of observables is limited and the parameters of the model are not known. Furthermore, the observables (eigenmode amplitudes and phases) vary in a non-trivial fashion, as illustrated in Figure 11a, Figure 12a and Figure 13a for a range of values of *K*_inf_, *K*_0_ and *C*_diss_, respectively. As the results suggest, it is possible for the trends to be non-monotonic and non-smooth, such that simple mappings of these observables do not provide an accurate picture of the variation of material properties across the sample (see comment in the captions of Figure 11 and Figure 12 regarding the origin of the kinks in some of the traces). Additionally, Figure 11b, Figure 12b and Figure 13b suggest that the trends in dissipated energy and peak forces are not unique for each of the three SLS parameters. For example, an asymptotic decrease in the dissipated energy (Figure 11b and Figure 13b) can be caused by a linear increase in the magnitude of *K*_inf_ or *C*_diss_, or a combination of both. Similarly, increases in the peak tip–sample forces can be caused by increases in any of the three SLS parameters.

#### Compositional mapping based on average quantities

The quantities plotted in panels (b) of Figures 11–13 are important from the physics point of view, but are generally not directly observable in AFM. Instead, the user commonly relies on quantities calculated from the phases and amplitudes of the two active eigenmodes (in the case of AM-OL), namely the average calculated dissipated power (*P*_ts_) and the virial (*V*_ts_) for each of the modes [27,32,37]. These quantities are plotted in panels (c) and (d), respectively, in Figures 11–13 as a function of the SLS parameters, varying one parameter at a time (the calculated dissipated power was converted to energy per fundamental cycle in order to allow for a comparison with panel (b) of each figure). Inspection of these results suggests that there is no guarantee that the dissipated energy calculated from *P*_ts_ correlates with the energy that is dissipated due to the nature of the SLS model, calculated by integrating the area of the dissipation loops in the force–position trajectories (compare, for example, the dissipated energy plotted in Figure 12b with the two traces in Figure 12c). Furthermore, there is not always a strong correlation between the results for each of the eigenmodes (see Figure 11c and Figure 12c). Inspection of panels (b) and (d) of Figures 11–13 show that the correlation between *V*_ts_ and the peak tapping forces can also be weak or even non-existent. *V*_ts_ is expected to show a negative correlation with the relative importance of the ‘conservative interactions’ but these are not directly separable from the non-conservative interactions in the SLS model. Rather, they are coupled and there is not a straightforward physical interpretation of *V*_ts_. These discrepancies are not surprising in light of this coupling of conservative and dissipative interactions, in light of the non-trivial dissipation mechanism discussed above, which differs from a simple dissipation coefficient, and in light of the complexity of multi-frequency tip–sample interactions, which can lead to a variety of dynamics subtleties, even including severe artifacts such as contrast inversion depending on the imaging conditions [42,48–49].

## Discussion

As the results shown in the previous sections illustrate, the interactions of viscoelastic surfaces with the AFM tip can be extremely complex, especially if multi-frequency AFM methods are involved. The relatively large oscillations of the tip in intermittent-contact measurements, as well as the sharp variations in the forces make it necessary to use a tip–sample interaction model that includes the relevant types of attractive and repulsive interactions, as well as the intrinsic behaviors of viscoelastic surfaces, namely creep compliance, stress relaxation and a response that depends on the timescale of application of the stresses or deformations. Despite the simplicity of the SLS model, the richness of the observed phenomena can already seem to be overwhelming when one considers possible means to invert the cantilever response to obtain the surface model parameters. The use of more elaborate models, such as models with multiple relaxation times or nonlinear springs would further complicate matters and would make the inversion even more challenging since the number of observables in an experiment is limited unless the acquired data is enriched through procedures such as volume scanning [50–51]. Nonetheless, one can envision at least two research avenues that should prove very fruitful in this endeavor.

Firstly, the results presented here suggest that it would be advantageous, when characterizing viscoelastic samples, to carry out multiple measurements involving different timescales in order to capture the different relaxation times of the sample. In practice this can be accomplished by sequentially studying the same sample with cantilevers of different fundamental frequency or by using different higher eigenmodes in successive experiments. Even if an inversion methodology is not available, this approach can help to discern between samples of seemingly identical properties at a given timescale, especially if it is combined with volume scanning spectroscopy, which would provide depth-dependent information. Figure 5b, for example, shows that for constant-indentation imaging of the sample with increasing higher mode amplitude (if such a method can be developed) the level of dissipation decreases monotonically, which is as expected, since the SLS has only one characteristic relaxation time (governed by the damper) and the sample deformation is transitioning from a low frequency deformation (governed by the fundamental mode) to a high frequency deformation (governed by the higher mode). However, a real sample may have more than one characteristic relaxation time, which could be probed by gradually increasing the amplitude of different higher eigenmodes in successive experiments, under constant indentation. The analysis could be repeated at different levels of indentation to give a complete picture of the depth-dependent behavior of the sample.

Second, the further development of spectroscopy methods that provide the tip–sample force curve for individual impacts [25–26,52–53] with high accuracy will be extremely beneficial, as it can allow expanded representations of the tip–sample force dependence (for example, representations in which the force is measured with respect to not only position but also velocity [10]). The acquisition of such signatures of the tip–sample interaction force can in turn spur the development of inversion methodologies for different types of models. An example of this type of inversion for an SLS surface is offered in reference [40]. This study demonstrates mathematically that such an inversion is possible even with conventional force–distance curves (force vs distance), although it assumes very high accuracy in the acquisition of the tip–sample force curve through the spectral inversion method [26]. In practice there exist limitations that preclude such accuracy [38]. The development of new real-time spectroscopies remains at the forefront of multi-frequency AFM research [9,26,53–55], although to the knowledge of the author there is not yet a single-cycle, model-free experimental spectroscopy technique that can provide a sufficiently high accuracy to reproduce the sharp turns in the tip–sample force curve, especially around the force minimum dictated by the maximum attractive force. Besides the above two research avenues, further studies on realistic 3D contact models within the specific context of the most recently developed AFM techniques would also be extremely beneficial. In general, a detailed treatment of viscoelasticity within the ever-growing of number of intermittent-contact AFM techniques is in the opinion of the author an extremely important area of opportunity necessitating a combination of strong experimental, analytical and computational efforts.

## Conclusion

A numerical simulation study of the interactions observed in single-mode and bimodal AFM characterization of viscoelastic surfaces modeled as standard linear solids (SLSs) is presented. Examples of the extremely complex behavior of the tip–sample forces and observables are provided, along with an illustration of their dependence on the surface model parameters. The SLS model is the simplest that can reproduce stress relaxation and creep compliance, which are important fundamental behaviors observed in real viscoelastic surfaces. Additional opportunities remain in modeling real surfaces, which may require non-linear springs and multiple relaxation times. However, the inversion of the surface parameters from experimental data is an extremely challenging task even with the simple SLS. Further research is encouraged on single-cycle force spectroscopy measurement as well as inversion methodologies specific for viscoelastic surfaces.

### Methods

The numerical simulations of the cantilever dynamics were carried out including three eigenmodes of the AFM cantilever as in previous studies [24,42]. Active eigenmodes, as indicated throughout the paper, were driven at their natural frequency. The surface was modeled in most cases as a SLS (Figure 1a), except for the result of Figure 1c, which uses the combination of a Hertzian contact model with a depth dependent dissipation constant [34,42]. Long-range attractive interactions were included for a tip radius of curvature of 10 nm and a Hamaker constant of 2 × 10^−19^ J. In some cases, the oscillation of the tip was prescribed along ideal single-mode or bimodal trajectories, as indicated in the text, by relaxing only the SLS in real time. The cantilever force constant was set to *k* = 4 N/m and the first two quality factors to *Q*_1_ = 150 and *Q*_2_ = 450 in all cases (the frequencies varied and are provided along with the results). The amplitude and phase of each eigenmode, where applicable, were calculated by using the in-phase (*I*) and quadrature (Q) integrals:

[2]
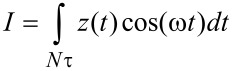


[3]
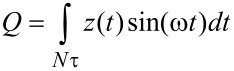


where *z*(*t*) is the eigenmode response in the time domain, *N* is the number of periods over which the phase and amplitude were averaged, ω is the excitation angular frequency, and τ is the nominal period of one oscillation. The amplitude and phase were calculated, respectively, as:

[4]
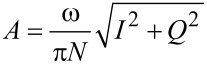


[5]
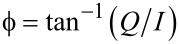


The average dissipated power (*P*_ts_) and virial (*V*_ts_) for panels (c) and (d) in Figures 11–13 were calculated as follows (panel (c) of these figures shows the energy dissipated per cycle, which was obtained by multiplying *P*_ts_ by the fundamental period) [30–32]:

[1]
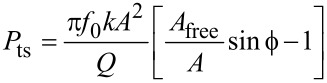


[6]